# Neuronal nitric oxide synthase expression in secretory cells of the human parotid and submandibular salivary glands: evidence from light and electron microscopy

**DOI:** 10.1007/s00441-026-04048-7

**Published:** 2026-02-13

**Authors:** Michela Isola, Marianna Boi, Roberto Demontis, Raffaella Isola, Francesco Loy, Cristina Maxia, Daniela Murtas, Maria Pina Serra, Jörgen Ekström, Marina Quartu

**Affiliations:** 1 Department of Biomedical Sciences, Section of Cytomorphology, University of Cagliari, Cittadella Universitaria Di Monserrato, 09042 Monserrato, CA Italy; 2 Department of Medical Sciences and Public Health, University of Cagliari, Legal Medicine Division ARNAS Brotzu, Cagliari, Italy; 3 Department of Pharmacology, Institute of Neuroscience and Physiology, The Sahlgrenska Academy at University of Gothenburg, Medicinaregatan 13, Box 431, 405 30 Göteborg, Sweden

**Keywords:** Secretory vesicles, Serous acini, Immunohistochemistry, Salivary glands, Neuronal Nitric oxide synthase

## Abstract

Nitric oxide plays a crucial role in multiple physiological processes in salivary glands, yet its precise cellular sources in human glandular tissues remain insufficiently characterized. This study investigated the distribution and subcellular localization of neuronal nitric oxide synthase (nNOS) in human parotid and submandibular gland tissue samples obtained from surgical biopsies and autopsies, using both light and electron-microscope immunohistochemistry. In both gland types, nNOS was consistently observed in serous secretory cells and the ductal epithelium, whereas mucous cells showed no immunoreactivity. Ductal cell staining was heterogeneous, with some cells displaying intense labeling and others remaining unstained. Nerve fibers containing the enzyme were rare within the gland parenchyma. Ultrastructural analysis revealed the enzyme within secretory granules and small cytoplasmic vesicles of serous cells, as well as in the apical and basal cytoplasm, mitochondria, and nuclei of ductal epithelial cells. These findings provide the first ultrastructural evidence of nNOS in secretory and ductal cells of human major salivary glands. The selective localization in serous cells, absence in mucous cells, and limited nitrergic innervation suggest a substantial parenchymal contribution to nitric oxide production. This distribution indicates that nitric oxide regulates saliva formation, composition, and intracellular signaling and could play a broader role in oral physiology and disease.

## Introduction

Nitric oxide (NO) has been implicated in a range of salivary glandular activities. In both animal and human salivary glands, nitrergic nerves are distributed to varying extent near acini, ducts, and blood vessels, as demonstrated by immunochemical detection of neuronal nitric oxide synthase (nNOS) and histochemical identification of its co-factor, nicotine adenine dinucleotide phosphate-diaphorase (NADPH-d). This nitrergic innervation is of parasympathetic origin (Lohinai et al. 1995; Soinila et al. 1996; Alm et al. 1995, 1997). Animal studies involving cats, ferrets, and pigs show that stimulation of parasympathetic innervation of the submandibular gland enhances salivary secretion and glandular blood flow through NO-mediated mechanisms and that these effects are partly due to the promotion of vasoactive intestinal peptide (VIP) release from parasympathetic nerve terminals and partly to postjunctional actions of VIP, which involve NO generation in effector cells (Modin, 1994; Edwards, 1998; Tobin et al. 1997). At the target level, NO acts intracellularly by increasing cyclic guanosine monophosphate (cGMP) in response to activation of soluble guanylate cyclase as observed in acinar cells of the rat parotid gland (Looms et al. 2000). Additional functional studies, mainly using the rat parotid gland as experimental model, support the role of glandular parenchyma as a source of NO, contributing to amylase and protein secretion, protein synthesis, and mitotic activity in response to specific receptor agonists (Sayardoust & Ekström, 2003; Ekström et al. 2007; Aras & Ekström, 2008; Çevik-Aras et al. 2011). As judged by reduced responses in the presence of the highly selective nNOS inhibitor N-propyl-L-arginine (N-PLA) (Zhang et al. 1997). Notably, N-PLA is as effective as the non-selective NO synthesis inhibitor N-omega-nitro-L-arginine methyl ester (L-NAME), which blocks not only nNOS, but also endothelial NO-synthase (eNOS) and inducible NO-synthase (iNOS), suggesting a seemingly exclusive involvement of the neuronal type of NOS (Moncada 1999). Regarding the cellular source of NO, reports are at hand for just a few animal species, with focus on the rat but also including observations, using light microscopy, on the cat, ferret, and mouse (Issy et al. 2009; Jeong et al. 2000; Alm et al. 1997; Soinila et al. 1996; Lomniczi et al. 1998; Lohinai et al. 1995, 1997; Ambe et al. 2016). Acinar cells generally lack staining for nNOS and NADPH-d. In contrast, ductal elements predominantly exhibit NADPH-d, while nNOS may be absent. Vascular endothelium typically lacks nNOS staining, although NADPH-d may be present.

In humans, there are limited reports regarding the nitrergic innervation of the salivary glands, the involvement of NO-mediated mechanisms in glandular functions, and the potential cellular sources of NO. Isolated acinar cells from human labial glands exposed to noradrenaline responded with increased NOS activity, as indicated by an increase in the fluorescence intensity of the NO indicator DAF-2 (Looms et al. 2000). The three major salivary glands, as well as labial glands, generally display scarce to absent nitrergic innervation of secretory elements and blood vessels (Konttinen et al. 1997; Pedersen et al. 2018; Soinila et al. 2006; Boi et al. 2024). Consistent with animal studies, Soinila et al. (2006) found no clear-cut nNOS-labeling in acinar cells of the human salivary glands, whereas the ductal epithelium showed variable staining intensity, with some ductal cells lacking staining entirely. Vascular endothelium also lacked nNOS reactivity. Furthermore, Konttinen et al. (1997) reported strong NADPH-d activity in the myoepithelial cells and ducts of labial glands from patients with Sjögren’s disease.

Given the limited number of studies on human salivary glands and the lack of ultrastructural investigations of nNOS, further research may uncover previously unrecognized roles for this enzyme at the subcellular level. The parotid gland consists of serous cells, whereas the submandibular gland contains both serous cells and, to a lesser extent, mucous cells. Immunohistochemical analyses at both the light and electron microscopy levels have revealed patterns of nNOS immunoreactivity within secretory granules and ductal cells, suggesting possible morphological bases for the function of NO in salivary glandular activities.

## Materials and methods

### Sampling

Specimens of human major salivary glands, including 5 parotid and 5 submandibular samples were collected at surgery from ten patients, aged 60–65 years, undergoing radical excision for oncological pathologies involving the mouth and neck regions, who gave their informed consent to participate in the research; further, 2 parotid and three submandibular gland samples were obtained at autopsy from five subjects, with no signs of oral or neurological pathologies, aged from 20 to 60 years. In the case of surgical samples, only glandular fragments with a size of 2–3 mm^3^ not compromised by the tumor or other oral pathologies were selected. Gland eligibility was confirmed by light (LM) and transmission electron microscopy (TEM) evaluation. Patients were not habitual smokers, alcohol consumers, or obese. Moreover, they were not affected by severe oral disorders and had not been subjected to chemotherapy or radiotherapy before surgery. In compliance with the principles laid out in the Declaration of Helsinki, the study was approved by the Independent Ethics Committee (IEC) of the Azienda Ospedaliero-Universitaria of Cagliari, Italy (Prot. PG/2019/10454 – Part 2.21). The IEC has formally stated the moral principles to which the present study adheres. All data were de-identified before database creation and data analysis. Tissues obtained from surgeries, to be processed for immunohistochemistry, were immediately fixed by immersion in phosphate-buffered 10% formaldehyde, pH 7.3 for 2 h, rinsed overnight (O.N.) in phosphate-buffered saline (PBS), and paraffin-embedded. Autoptic specimens were fixed by immersion in the same fixative for 4 h, rinsed O.N. in PBS, and processed either for paraffin embedding or cryostat sectioning.

### Light microscope immunohistochemistry

Microtome paraffin Sects. (6–7 µm thick) and cryostat Sects. (14 µm thick) of both biopsy and autopsy specimens were collected on chrome alum-gelatin-coated slides and processed using the avidin–biotin-peroxidase complex (ABC) immunohistochemical technique (Loy et al. 2020). Endogenous peroxidase activity was blocked using 0.1% phenylhydrazine (Cat# 101,326,606, Sigma-Aldrich, St. Louis, MO, USA) in PBS containing 0.2% Triton X-100 (PBS/T). In the case of paraffin-embedded tissue, antigen retrieval was accomplished by heating the samples at 90 °C for 20 min in 10 mM citrate buffer (pH 6.0), then allowing them to cool gradually for 20 min. The sections were then incubated with 20% normal goat serum (Cat #S-1000, Vector, Burlingame, CA, USA) followed by a rabbit polyclonal antiserum raised against recombinant human nNOS, which recognizes human, mouse, sheep, rat, and monkey nNOS but does not cross-react with eNOS or iNOS in immunohistochemistry (RRID:AB_91824; Cat #AB5380, Merck), diluted 1:1000 at 4 °C overnight. Then, the slides underwent incubation with a biotin-conjugated goat anti-rabbit IgG (RRID: AB_2313606; BA-1000, Vector, Burlingame, CA, USA), diluted 1:400, for 40 min at RT. The reaction product was revealed using the ABC (Cat#G011-61, BioSpa Div., Milan, Italy), diluted 1:250, followed by incubation in a solution of 0.1 M PB, pH 7.3, containing 0.05% 3,3′-diaminobenzidine (Sigma-Aldrich, St. Louis, MO, USA), 0.04% nickel ammonium sulfate, and 0.01% hydrogen peroxide. All antisera and the ABC were diluted in PBS/T. To verify antibody specificity, control immunostainings were performed in parallel, either with the same diluted primary antibody preabsorbed with recombinant human nNOS (Cat#MBS1573804, MyBiosource, San Diego, CA, USA) for 24 h at 4 °C, or by omitting the primary antisera, or by using PBS/T alone. These procedures resulted in a complete absence of immunostaining in the examined tissues. Some representative slides were counterstained with Carazzi’s hematoxylin after immunostaining. The slides were observed using an Olympus BX61 microscope, and digital images were captured with a Leica DFC450C camera.

### Transmission electron microscope immunohistochemistry

Bioptic samples of the parotid and submandibular glands were cut into small pieces and fixed for 2 h at room temperature using a mixture of 3% paraformaldehyde and 0.1% glutaraldehyde in 0.1 M cacodylate to preserve tissue antigenicity. Following fixation, the samples were rinsed in cacodylate buffer containing 3.5% sucrose and then dehydrated and embedded in Epon Resin (Glycide Ether100, Merck, Darmstadt, Germany). To further preserve antigenicity, osmium tetroxide treatment, commonly used as a stain for lipids in membrane organelles and vesicles, was omitted. Semithin Sects. (2 µm) of these tissue samples were stained with toluidine blue and examined using light microscopy to assess the histological appearance. Ultrathin Sects. (70–80 nm) were cut with a diamond knife, collected on nickel grids, and processed for immunohistochemical analysis as previously described (Isola et al. 2024). For labeling, grids were floated on 25–30 μl drops of reagent in Parafilm-lined petri dishes. Ultrathin sections were treated with 1% bovine serum albumin (BSA) and 5% normal goat serum (SIGMA, St. Louis, MO, USA) in PBS solution to block non-specific binding. They were then incubated overnight at 4 °C with the same rabbit polyclonal antiserum used for the light microscope immunohistochemistry, diluted 1:40 in PBS + 1% BSA and 5% NGS. The ultrathin sections were then incubated for 60 min at room temperature with the secondary antiserum, a goat anti-rabbit IgG conjugated to 15-nm gold particles (GE Healthcare, Chalfont St. Giles UK), diluted 1:50 in PBS with 1% BSA. Following a wash with PBS, the ultrathin sections were stained with uranyl acetate and bismuth subnitrate and then observed and photographed using a JEOL JEM 1400 Plus (Center for Research University Services—CeSAR core facilities, University of Cagliari, Italy) and a JEOL 100S transmission electron microscope. Negative control immunostaining was performed by either incubating the sections with non-immune serum or omitting the primary antibody.

## Results

### Light microscope immunohistochemistry

**Fig. 1:**
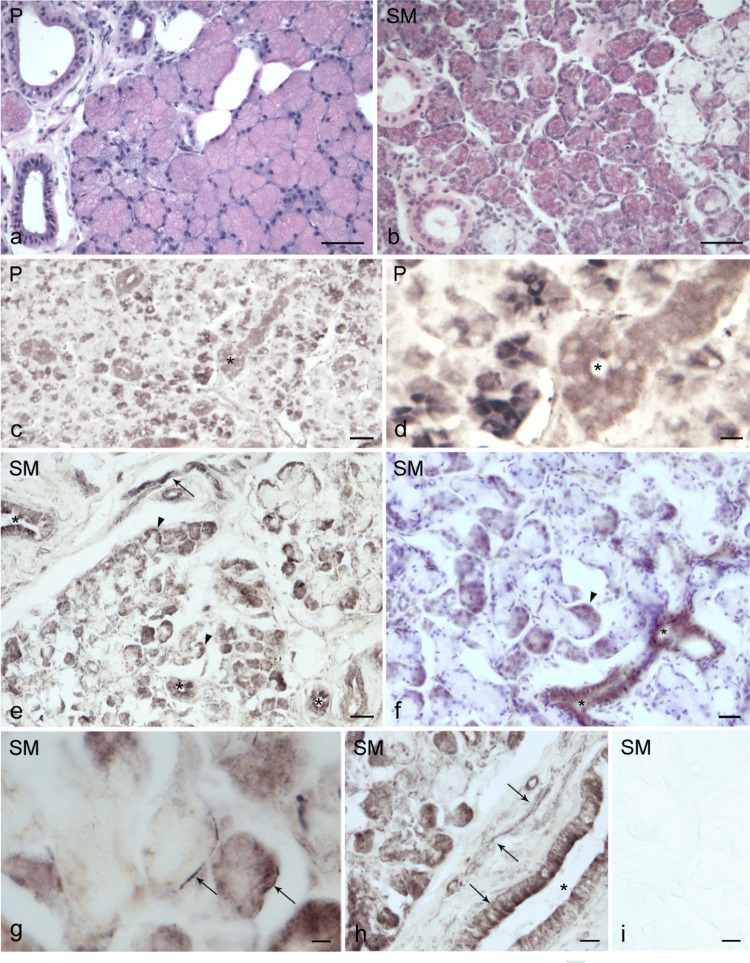
Light microscopy of nNOS immunostaining in human salivary glands. **a**, **b** Parotid gland (P); **e**–**h** Submandibular gland (SM). nNOS immunolabeling is present in serous acinar cells of the parotid (**c**, **d**) and submandibular glands (**e**–**h**), as well as in submandibular serous demilunes (**e**, **f**) (arrowheads) and ducts (**c**–**f**, **h**) (asterisks). nNOS-immunostained nerve fibers (arrows) are observed as bundles of varying caliber running in the stroma (**e**), as individual fibers (**e**), or as coarse terminals near serous (**d**, **g**) and mucous (**g**) secretory cells, or as arrays of thin dot-like terminals placed against the duct walls (**h**). **a**, **b** Hematoxylin and eosin staining and **f** Carazzi’s hematoxylin counterstaining. **i** Negative control for immunostaining. Scale bars: 100 µm (**a**, **b**, **c**, **e**); 50 µm (**d**, **f**, **h**, **i**); 20 µm (**g**)

All examined parotid and submandibular gland samples consistently showed nNOS-immunostaining (Fig. 1). The immunolabeling was present in both nervous structures and non-nervous structures. Non-nervous elements included serous secretory cells and ductal components associated with the serous acini, ranging from striated and intercalated to large excretory ducts (Fig. 1). No nNOS-immunostained mucous cells were observed in the submandibular gland. Furthermore, no specific nNOS-immunostaining was detectable in the endothelial lining of blood vessels. Mild-to-strong nNOS-immunolabeling appeared in the cytoplasm of serous cells within acini of both glands and in the crescent-shaped formations (demilunes) of serous cells that cap mucous alveoli in the submandibular glands (Fig. 1). In contrast, ductal epithelial lining displayed patchy nNOS-immunolabeling, with strongly positive cells adjacent to negative ones (Fig. 1). Occasionally, nNOS-positive nerve fibers were seen in the parenchyma, presenting as short filaments or punctate elements running near both mucous and serous secretory cells, ducts, and blood vessels (Fig. 1). Nerve fiber bundles of different calibers were also present in the interlobular connective tissue (Fig. 1). Negative control samples lacked nNOS-immunolabeling (Fig. 1g).

### Transmission electron microscope immunocytochemistry

**Fig. 2:**
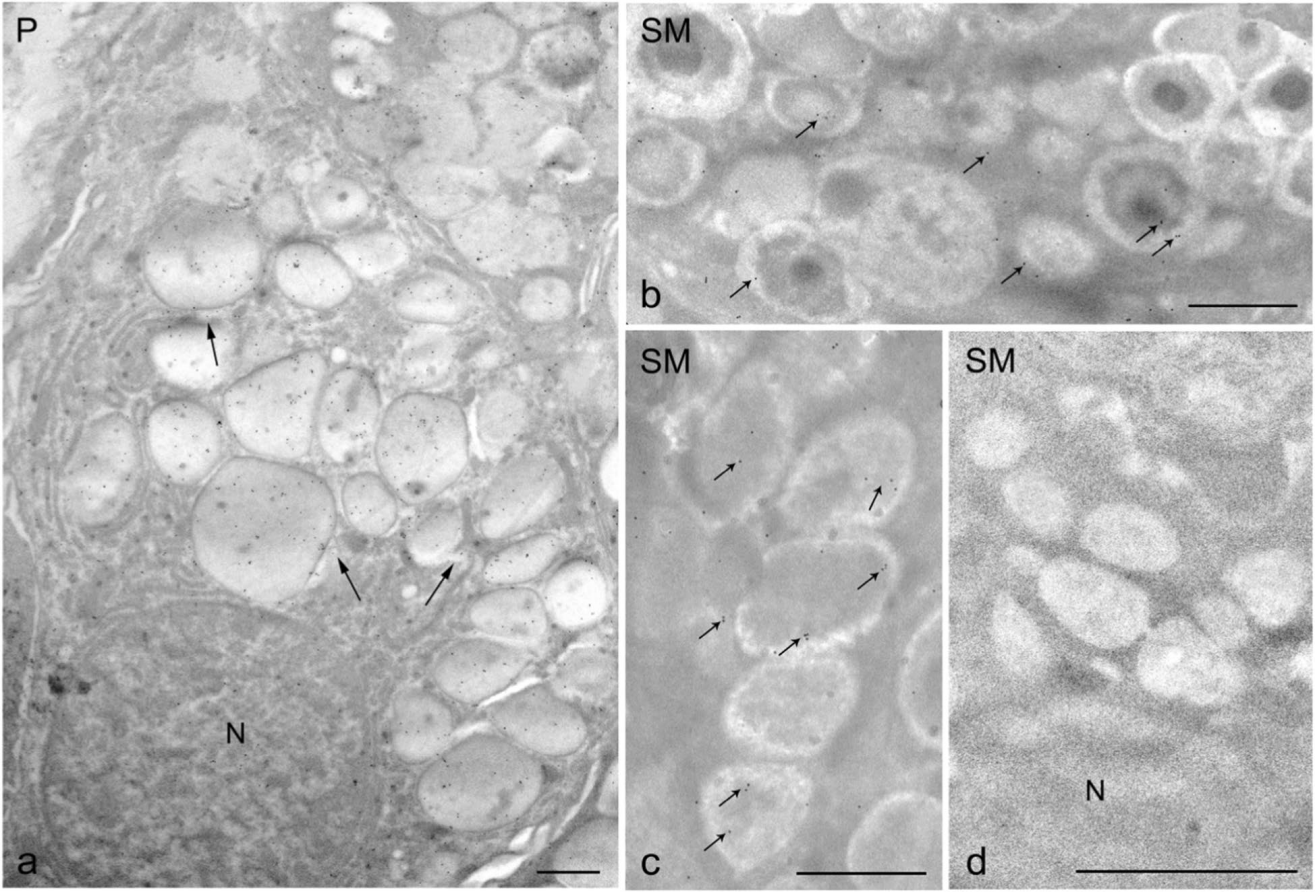
Transmission electron microscopy of nNOS immunostaining in human salivary glands, visualized using the immunogold technique (**a**–**d**). **a** A portion of a parotid (P) serous cell shows immunogold labeling within secretory granules. Small cytoplasmic vesicles (arrows) among the granules also display labeling, and the nucleus of the serous cell contains gold particles. **b**, **c** High-magnification images of submandibular gland (SM) serous cells demonstrate immunolabeled secretory granules (small arrows). **d** In contrast, mucous droplets in submandibular gland cells are unlabeled. N, nucleus. Scale bars 1 µm (**a**–**d**)

**Fig. 3:**
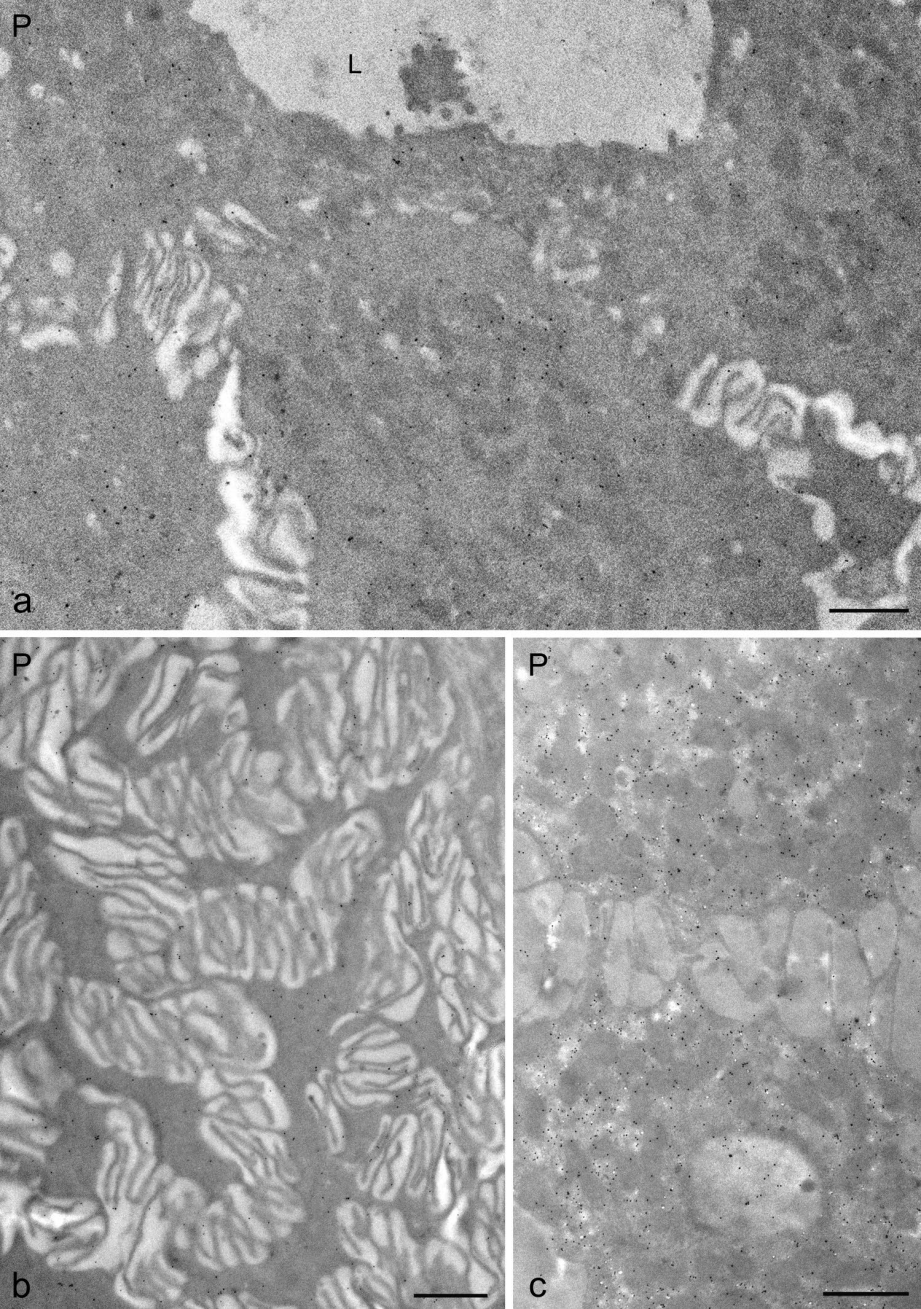
Transmission electron microscopy of nNOS immunostaining in human salivary glands, visualized using the immunogold technique (**a**–**c**). **a** High-magnification image of the apical portion of striated ductal cells in the parotid gland. **b**, **c** High-magnification details of the basal portion of striated ductal cells in the parotid gland, showing gold particles associated with basal folds and mitochondria. L, lumen; P, parotid. Scale bar 1 µm (**a**–**c**)

**Fig. 4:**
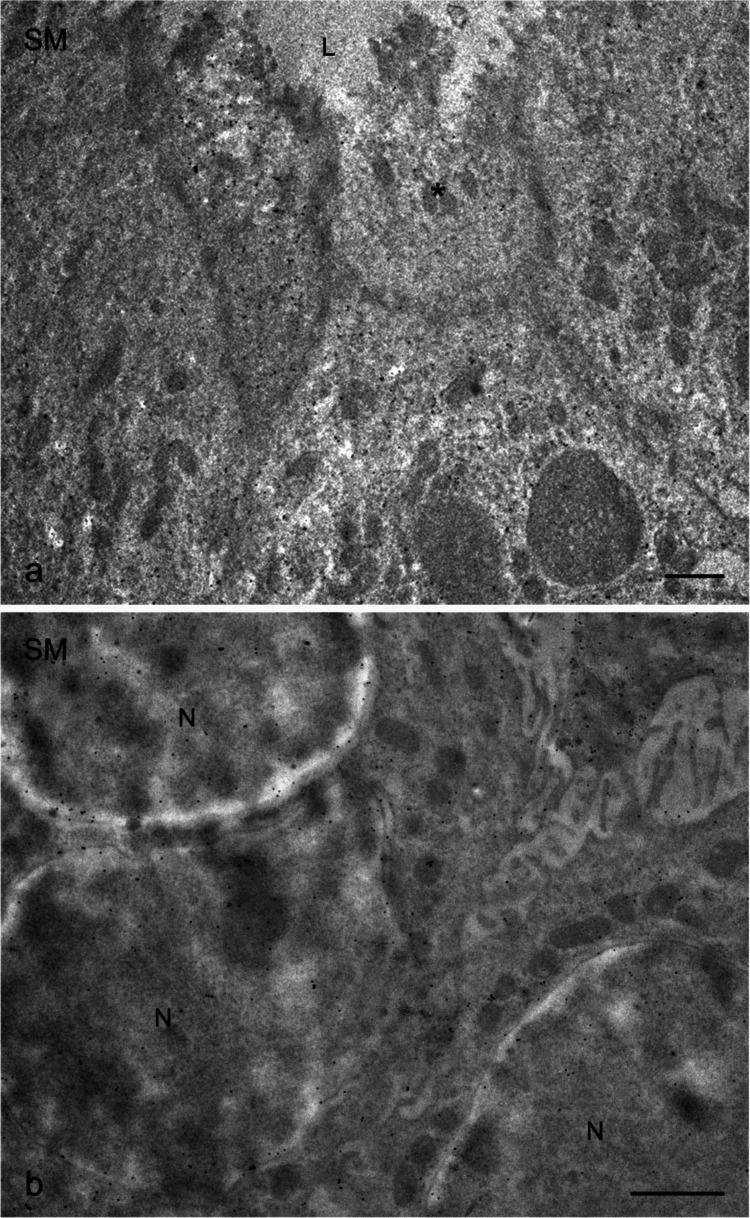
Transmission electron microscopy of nNOS immunostaining in human submandibular glands (SM), visualized using the immunogold technique (**a**, **b**). **a** High-magnification image of the apical portion of the epithelial lining of striated ducts, showing heterogeneous labeling. Some cells show sparse immunolabeling (asterisks). **b** Basal portion of striated ductal cells with immunogold labeling observed in mitochondria and nuclei. N, nucleus. Scale bar 1 µm (**a**, **b**)

The immunogold labeling consistently revealed the presence of nNOS in multiple cellular components of both the parotid and submandibular glands (Figs. 2, 3, 4).

Strong nNOS immunolabeling was primarily detected in the cytoplasm of serous secretory cells and ductal cells. Immunogold particles were particularly concentrated within the secretory granules in the apical regions of serous cells (Fig. 2) and small cytoplasmic vesicles among these granules also exhibited labeling (Fig. 2). Nuclei contained gold particles as well (Fig. 2). Notably, mucous cells of the submandibular gland lacked labeling (Fig. 2d), indicating a selective distribution of nNOS in serous, but not mucous, cell types.

Within the ductal system, including intercalated and striated ducts, nNOS labeling was present in both apical and basal regions of ductal epithelial cells. In striated duct cells, gold particles were frequently associated with basal folds, mitochondria, and nuclei (Figs. 3 and 4). Interestingly, mirroring the findings at the light microscope level, some striated duct cells exhibited only sparse immunogold labeling, while others were decorated with a multitude of gold particles (Fig. 4). Nuclei of ductal cells also showed immunogold labeling (Fig. 4). No gold particles were observed in the vascular endothelium.

**Fig. 5:**
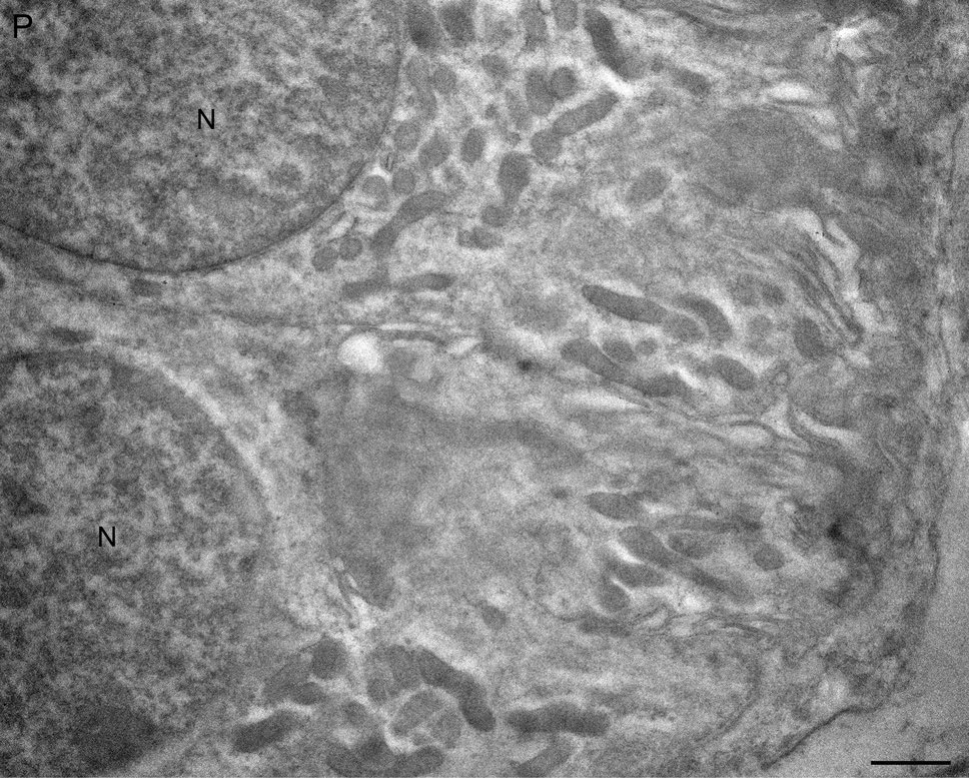
Human parotid gland (P). Negative control for immunostaining. N, nucleus. Scale bar 1 µm

No immunolabeling was detected in the control samples, confirming the specificity of the antibody used (Fig. 5).

## Discussion

This study demonstrates nNOS-immunoreactivity in acinar serous cells and throughout the intercalated and excretory ductal branches of all examined salivary gland samples at both the light microscopic and ultrastructural levels, underscoring the potential role of nNOS in salivary flow/composition and/or in the regulation of salivary secretion. Our findings are only partially consistent with previous studies, which reported that nNOS-immunoreactivity is predominantly localized in ductal cells (Soinila et al. 2006; Asakawa et al. 2015).

In agreement with earlier reports, the vascular endothelium of salivary glands lacks immunoreactivity for nNOS (Soinila et al. 2006).

Our results expand current knowledge by providing the first ultrastructural evidence of nNOS association with cytoplasmic organelles in human salivary gland acinar and ductal cells. The presence of nNOS-immunoreactivity within apical granules of serous acinar cells supports the concept that the role of NO in secretory activity is not exclusively linked to innervation but may also originate from parenchymal sources (Looms et al. 2002; Sayardoust & Ekström, 2003; 2004), consistent with earlier hypotheses (Bodis & Haregewoin 1993). Localization of nNOS near or within secretory vesicles suggests that NO is generated adjacent to the exocytosis machinery, where it acts as a signaling molecule in vesicle fusion with the plasma membrane.

The lack of nNOS immunoreactivity in mucous cells of the submandibular gland observed in the present study is consistent with previous light microscopy findings (Asakawa et al. 2015; Soinila et al. 2006). There is no ready explanation for the differential distribution of nNOS between serous and mucous secretory cells in the submandibular gland. The selective localization of nNOS to serous cells suggests a tightly regulated and compartmentalized production of nitric oxide (NO). In contrast, mucous cells, which are specialized for the secretion of glycoprotein-rich mucins, appear to lack intrinsic nNOS expression. This uneven distribution likely reflects fundamental differences in the secretory roles and regulatory mechanisms of these cell types. The presence of nNOS in serous cells, which produce watery, enzyme-rich fluids, supports a direct role for NO in modulating serous secretion. In contrast, the absence of nNOS in mucous cells raises the possibility that their secretory responses are either independent of NO or modulated indirectly via paracrine signaling from nNOS-expressing nerve fibers or neighboring serous cells. The anatomical proximity of mucous cells to nNOS-positive structures supports the possibility of indirect modulation, although the functional consequences of such interactions remain unclear.

In the literature, little attention has been paid to NO’s role in regulating salivary mucin secretion. The only study conducted in salivary tissue reports that the administration of an NO donor decreases mucin synthesis in isolated mucous acinar cells from the rat sublingual gland (Slomiany & Slomiany 2002), providing limited evidence of a stimulatory role for NO in salivary mucous cells.

Evidence from non-salivary tissues indicates that the impact of NO on mucin secretion is context-dependent. In the rat gastric mucosa, mucosecretory cells display nNOS immunoreactivity (García-Vitoria et al. 2000), and, in response to NO generation associated with elevated cGMP, mucin is secreted in the rat stomach (Brown et al. 1992, 1993). However, in the airway submucosal glands, the effects of NO vary by species and experimental conditions: in rats, the NO/cGMP pathway does not affect mucin secretion, while the cAMP pathway is stimulatory (Bredenböker et al. 2001); in ferrets, NO inhibits secretion (Ramnarine et al. 1996); and in cats, NO stimulates secretion (Nagaki et al. 1995). Collectively, the diversity of NO’s effects across tissues and species precludes extrapolation to the human submandibular gland. Consequently, further experimental studies are required to elucidate whether and how NO participates in the regulation of mucin production in salivary glands and to determine if such effects occur through direct action on mucous cells or via paracrine mechanisms.

Notably, nNOS is transcribed into at least four alternative splicing variants, each associated with a different subcellular compartment. nNOSb generally shows a cytosolic localization, as reported in neuronal cells and myocytes (Percival et al. 2010), while nNOSa and nNOSm are mostly associated with the plasma membrane (Brenman et al. 1996) but can also be recruited to the nucleus and activate nNOS-dependent mitochondrial biogenesis (Aquilano et al. 2014). The present study identified nNOS immunoreactivity in both the nuclei and mitochondria of serous cells and striated ducts. Although mitochondrial NOS, thought to be involved in mitochondrial respiration, has been tentatively suggested to correspond to the splicing variant nNOSa (Ghafourifar & Cadenas 2005; Tengan & Moraes 2017), there are currently no reports on the presence of nNOS splicing variants in salivary glands. The uneven nNOS immunolabeling observed may suggest that different splicing variants and thus distinct intracellular roles contribute to the modulation of saliva components or reflect different temporal patterns of secretion. Various cell membrane receptors, as indicated by animal studies, may be involved in the intracellular mobilization of NO, including β-adrenoceptors, VIP-receptors, muscarinic receptors, and receptors for gastrin and melatonin. However, the evidence is inconsistent, possibly reflecting species and gland differences or contradictory findings for one and the same gland (see Looms et al. 2002; Ekström et al. 2004; Cevik Aras & Ekström, 2006; Aras & Ekström, 2008; Sayardoust & Ekström, 2003, 2004; Cevik Aras & Ekström 2006; 2008; Çevik-Aras et al. 2011; Issy et al. 2009; Rosignoli & Pérez Leirós 2002; Sugiya et al. 2001; Takai et al. 1999). While the intracellular action of NO in salivary glands is usually associated with cGMP formation and exocytotic events (Looms et al. 2002), another intracellular pathway for NO via S-nitrosylation, in which NO reacts with cysteine thiols to regulate a number of processes, including transcription, cellular growth, and cell differentiation, is known. This second pathway has not yet been explored in salivary glands (Horenberg et al. 2019; Fernando et al. 2019), in contrast to pancreatic acinar cells (Chvanov et al. 2006).

Some animal studies have demonstrated NADPH-d, but not nNOS immunoreactivity, in the ducts (Harrison 1974; Alm et al. 1997; Singh & Singh 2016). Although NADPH-d activity is associated with nNOS-immunoreactivity in neuronal tissue, its activity in epithelial cells does not necessarily indicate the presence of nNOS (Lohinai et al. 1995). Therefore, it is notable that nNOS immunoreactivity was demonstrated in ducts in the current study, consistent with Soinila et al. (2006).

Ultrastructural analysis revealed that nNOS localizes to the apical cytoplasm and mitochondria of striated ductal cells, as well as to the apical cytoplasm of intercalated ducts. The physiological significance of cytosolic nNOS in ductal cells remains to be clarified, although NO in saliva may contribute to the natural antibacterial properties of salivary secretion and to the detoxification of potential oral carcinogens (Bodis & Haregewoin 1993). Given the limited nitrergic innervation of the human salivary gland parenchyma, it has been proposed that NO may act in a paracrine fashion (Soinila et al. 2006), influencing neighbouring cells and potentiating nerve stimulation (see also Looms et al. 2000). The distinct nNOS-immunolabeling observed in serous versus mucous cells suggests that duct cell differences may reflect their association with different secretory elements; however, further studies are required to confirm this inference.

Interest in NO has increased substantially since its identification, and a deeper understanding of NO in oral tissues may advance the treatment of oral diseases. Although the functions of NO in oral diseases remain underexplored, elevated salivary nitrite levels have been associated with oral pathologies, including periodontal disease, oral inflammatory disease, and tumor invasiveness in salivary gland cancers. These associations provide a rationale for exploring the therapeutic potential of modulating NO production through specific NOS inhibitors in salivary glands (Brennan et al. 2003). Decreased nNOS in submandibular glands correlates with dry mouth in diabetes mellitus (Stewart et al. 2016), while increased NO and nitrosative stress are observed in patients with plaque psoriasis (Skutnik-Radziszewska et al. 2020).

## Data Availability

All data supporting the findings of this study are available from the corresponding author (Marina Quartu, mailto:quartu@unica.it) upon request.

## Abbreviations

PBS: Phosphate-buffered saline
ABC: Avidin–biotin–peroxidase complex
nNOS: Neuronal nitric oxide synthase
